# The role of the cell–cell interactions in cancer progression

**DOI:** 10.1111/jcmm.12408

**Published:** 2015-01-19

**Authors:** Katarzyna Kamińska, Cezary Szczylik, Zofia F Bielecka, Ewa Bartnik, Camillo Porta, Fei Lian, Anna M Czarnecka

**Affiliations:** a Department of Oncology with Laboratory of Molecular Oncology, Military Institute of MedicineWarsaw, Poland; b Postgraduate School of Molecular Medicine, Medical University of WarsawWarsaw, Poland; c Institute of Genetics and Biotechnology, Faculty of Biology, University of WarsawWarsaw, Poland; d Institute of Biochemistry and Biophysics, Polish Academy of SciencesWarsaw, Poland; e Medical Oncology, I.R.C.C.S. San Matteo University Hospital FoundationPavia, Italy; f Emory University School of MedicineAtlanta, GA, USA

**Keywords:** cancer, tumour microenvironment, cell–cell interactions, stromal cells

## Abstract

In the field of cancer research, scientific investigations are based on analysing differences in the secretome, the proteome, the transcriptome, the expression of cell surface molecules, and the deregulation of signal transduction pathways between neoplastic and normal cells. Accumulating evidence indicates a crucial role in carcinogenesis concerning not only stromal cells but also normal cells from target organs and tissue where tumours emerge. The tumour microenvironment (TME) definitively plays an important role in regulating neighbouring cell behaviour. To date, limited attention has been focused upon interactions between cancer cells and normal cells. This review concentrates on the interactions between stromal and healthy cells from the TME in cancer development. In the article, the authors also describe mutations, genes and proteins expression pattern that are involved in tumour development in target organ.

- Introduction
- Cancer cell – fibroblast interaction in cancer progression
- Cancer cell – hepatocyte interaction in cancer progression
- Cancer cell – lung epithelial cell interaction in cancer progression
- Interaction between cancer cells and normal cells of the primary organ
- Cancer cell – pleural and peritoneal cells interaction in cancer progression
- Summary

## Introduction

Cell populations that assist haematopoietic stem cells and its progeny are called stromal cells. *In vitro,* these cells form non-haematopoietic adherent cell components from long-term cultures and *in vivo* make up the microenvironment of haematopoiesis, comprising the set of non-haematopoietic cells from the different haematopoietic sites 1. Similarly, tumours have ‘their’ stromal cells which consist of non-malignant cells of the tumour such as cancer-associated fibroblasts (CAFs), specialized mesenchymal cell types characteristic to each tissue environment, innate and adaptive immune cells, vasculature with endothelial cells and pericytes, the extracellular matrix (ECM) consisting of structural proteins (collagen and elastin), specialized proteins (fibrillin fibronectin and elastin) and proteoglycans 2.

Research indicates that the cell environment profoundly affects cancer development. Moreover, it has confirmed the Stephen Paget's ‘seed and soil’ theory from 1889. He postulated that metastases of a particular type of cancer (‘the seed’) often metastasizes to certain sites (‘the soil’) based on the similarity of the environments of the original and secondary tumour sites 3. Present studies confirm this theory and reveal that the tumour microenvironment (TME) is the mentioned ‘soil’ 4–7. In carcinogenesis and cancer spread, TME determines the underlying processes. According to the National Cancer Institute, TME is described as ‘the normal cells, molecules, and blood vessels that surround and feed a tumour cell; a tumour can change its microenvironment, and the microenvironment can affect how a tumour grows’. Hallmarks of cancer, such as deregulated ECM, continually activated proliferative signalling, inhibition of suppressors and apoptosis, activating invasion and metastasis, deregulated of cell energetics, and abrogation of immune destruction are mostly regulated by TME. In addition, primary tumours secrete factors that alter the microenvironment of distant organs, making them suitable target for subsequent metastatic cancer cell colonization. The non-malignant cells of stromal tissue produce a unique microenvironment that can modify the neoplastic properties of the tumour cells 8. The now-increasingly accepted importance of TME, is embodied in the concept that cancer cells do not manifest the disease just by themselves, but rather conscript and corrupt resident and recruited normal cell types 9. The niche, or local microenvironment, of a cancer cell plays an important role in tumour progression.

Hanahan *et al*. (2012) proposed the division of the TME stromal component into three general classes: (*i*) angiogenic vascular cells, (*ii*) infiltrating immune cells and (*iii*) cancer-associated fibroblastic cells. These phenotypically, oncologically and morphologically different cell populations interact with each other to ultimately contribute to cancer cells growth, local invasion and metastasis. Fibroblasts are the predominant cells of the stroma and several publications have reported genetic and epigenetic changes in stromal fibroblasts that modulate the expression of many genes encoding growth factors and cytokines 10,11. Fibroblasts produce *inter alia* ECM molecules such as fibronectin and tenascin, which influence both cell adhesion and proliferation 8.

It also bears mentioning that mammalian genomes include a considerable number of endogenous retroviruses (ERVs). These relics of ancestral infectious retroviruses resulted from ancestral germ line infections by exogenous retroviruses which have thereafter been transmitted in a Mendelian fashion. Almost 8% of the human genome comprises ERVs 12. By analogy to exogenous tumourigenic retroviruses, ERVs have been implicated in the pathogenesis of cancer. Several viruses are linked with cancer in humans. Viruses are responsible for 18% of cancers worldwide 13. Many individuals are infected with viruses which may cause cancer, but usually without no symptoms. Not every infections develop into tumour which also confirms the theory that tumour cells are ‘picky’ about where they live.

A fundamental understanding of basic pathophysiological processes, for example malignant transformation, can in turn help to better define the targets for clinical intervention. As the cells and most factors from TME are well known, we focus on molecular interactions between healthy cells of the stroma and normal cells surrounding the tumour.

## Cancer cell–fibroblast interaction in cancer progression

Accumulating evidence indicates that CAFs play critical roles in cancer pathogenesis. CAFs are recruited from periacinar cells, circulating marrow-derived progenitors, vessel-associated pericytes, or other tissue-resident mesenchymal stem/progenitor cells 14,15. Myofibroblasts, a specialized type of fibroblast, are one of the predominant cell types in the cancer stroma and tend to aggregate peritumourally and encircle carcinoma cells invading adjacent normal tissue 16. CAFs have been intensively investigated and are a key component in both primary tumour development and metastasis 17,18.

The impact of CAFs reflects results obtained on a murine model of metastatic breast cancer 19. The authors revealed that fibroblasts from the cancer growth area are responsible for shift of the immune microenvironment from a Th2 to Th1 polarization. This modulation of immune polarization is associated with decreased tumour angiogenesis, lymphangiogenesis and suppression of spontaneous breast cancer metastasis. Elimination of CAFs suppresses spontaneous metastasis and enhances the anti-metastatic effects of chemotherapy 19. Indeed, studies of CAFs from renal cancer confirmed that cancer cells change cellular properties to support the invasion, migration and proliferation rate (AACR Annual Meeting 2014). Moreover, CAFs orchestrate tumour-related inflammation, tumour growth, invasion and angiogenesis 20–22. Observations from several cancer models indicate that the functional regulatory role of CAFs in tumourigenesis is defined *via* signalling pathways and mechanical stress 23–25.

Tumour growth stops at a diameter of about 1–2 mm unless sufficient blood supply develops 26,27. Tumour growth and progression depend on angiogenesis, a process of new blood vessel formation from a pre-existing vascular endothelium. Increasing evidence shows that CAFs are the major source of pro-angiogenic factors such as VEGF or PDGF 28,29. Signalling pathways activated by VEGF are considered to be crucial for angiogenesis 30, involving endothelial proliferation, survival, migration and the formation of vascular structures 31. PDGFs and their receptors (PDGFR) are involved directly and indirectly in angiogenesis. Indirectly, PDGF family members recruit VEGF-producing stromal fibroblasts 32. Moreover, tumour cells release pro-angiogenic factors into microenvironment resulting in secretion of PDGF by the endothelial cells, which attract supporting cells to stabilize the new vessel 33. Directly, PDGF released by neoplastic cells bind to their receptors on bone marrow progenitor cells following their recruitment and by signalling activation induce differentiation into endothelial or smooth muscle cells and promote both their growth and their migration 34,35.

The importance of CAFs in tumour progression is highlighted by the fact that they also act as major players in tumour metastasis. Transforming growth factor β (TGF-β) seems to be a major regulator of the processes in which CAFs are involved. TGF-β is a factor which causes recruitment of CAFs by cancer cells 36,37. Moreover, paracrine crosstalk between CAFs and cancer cells mediated by TGF-β signalling leads to an epithelial–mesenchymal transition (EMT) gain of cancer stem cell properties, which suggests that the CAFs contribute a specific niche for tumour progression 38.

Epithelial–mesenchymal transition is a process by which epithelial cells gain mesenchymal features causing cancer cell invasiveness, motility and cell stemness 39. Furthermore, EMT is presently considered one of the major resistance mechanisms to anti-angiogenic strategies. Indeed, in an elegant experiment, a biopsy of a cutaneous metastasis from a patient with clear cell renal cell carcinoma who initially responded to sunitinib, but ultimately progressed under therapy was grafted subcutaneously in athymic nude mice; established xenografts were treated with sunitinib and proved to be sensible again to its activity. More interestingly, histological examination of the original metastasis revealed evidence of EMT transition, whereas the xenografts showed reversion to the original clear cell phenotype. This way, Hammers *et al*. demonstrated that reversible EMT may be associated with acquired tumour resistance to the VEGF(Rs) tyrosine kinase inhibitor sunitinib in renal cell carcinoma 40.

*In vitro* studies have also shown that CAFs secrete matrix metalloproteinases MMP2, MMP9 and urokinase-type plasminogen activator (uPA) 41–43. The enhanced secretion of these proteolytic enzymes is believed to cleave various ECM components such as decorin, which covalently binds to TGF-β and consequently prevents the latter from binding to the TGF-β receptor in adjacent cancer cells and initiating EMT 44. Collectively, these observations suggest that both CAFs and myofibroblast niches may exert paracrine signalling regulation of epithelial phenotype plasticity 45.

The mesenchymal to amoeboid transition (MAT) process that follows EMT allows cells to glide through ECM barriers is also mediated by CAFs together with endothelial progenitor cells by bidirectional ephrinA1/EphA2 signalling 46. Thus, involvement of CAFs in these processes promotes cancer cell escape from the primary site and allows colonization of remote locations. Several observations indicate that inflammatory factors secreted by CAFs participate in this transformation and Wnt pathway 47–50. Interestingly, Duda *et al*. have provided evidence that the disseminating tumour cells metastasize to the secondary site together with passenger fibroblasts, from which early signalling originates that contributes to the pre-metastatic niche formation 51. In summary, CAFs not only are responsible for the orchestration of the above mentioned plastic changes of neoplastic cells but also for facilitating the formation of the metastatic niche.

Based on previously reported findings, Karagiannis *et al*. proposed a novel working model of metastatic growth progression based on both paracrine signalling and mechanical impact of the CAF cohort at the tumour–host cell interface 45. The ‘mechanical’ component of the proposed model postulates that CAFs migrate from cohorts that exert a mechanical pressure on the tumour invasion front, capable of changing the tissue-tension dynamics of the tumour cells population. Consequently, they may force the cancer cells to migrate towards less dense stromal regions 45.

The ‘paracrine’ component of the model postulates that CAFs may secrete paracrine soluble factors at the tumour invasion front, which may affect the various layers of the neoplastic population, generating phenotypic and signalling pathway gradients across the tumour cohort. CAFs may form niches similar to the ones occurring in various physiological and pathophysiological conditions in the human body 45. Indeed, CAFs from different cancer tissues manifest a pro-inflammatory gene signature including cyclooxygenase 2, osteopontin, chemokine ligand 1, chemokine (C-X-C motif) ligand 2, interleukin 6 (IL-6), IL-1β, chemokine (C-C motif) ligand 5 (CCL5), stromal-derived factor (SDF-1α) and tumour necrosis factor alpha 52–54. Among these factors, the SDF-1α is presently under active investigations in several cancer types.

Indeed, mesenchymal or marrow-derived stromal cells, which constitute a large proportion of the non-neoplastic cells within the TME, constitutively secrete SDF-1/CXCL12 chemokine. CXCL12 secretion by stromal cells attracts cancer cells, acting through its cognate receptor CXCR4, which is expressed by both haematopoietic and non-haematopoietic tumour cells. CXCR4 promotes tumour progression by direct and indirect mechanisms. First, CXCR4 is essential for metastatic spread to organs where CXCL12 is expressed and thereby allows tumour cells to access cellular niches that favour tumour cell survival and growth. Second, stromal-derived CXCL12 itself can stimulate survival and growth of neoplastic cells in a paracrine fashion. Third, CXCL12 can promote tumour angiogenesis by attracting endothelial cells to the TME 55.

**Fig 1:**
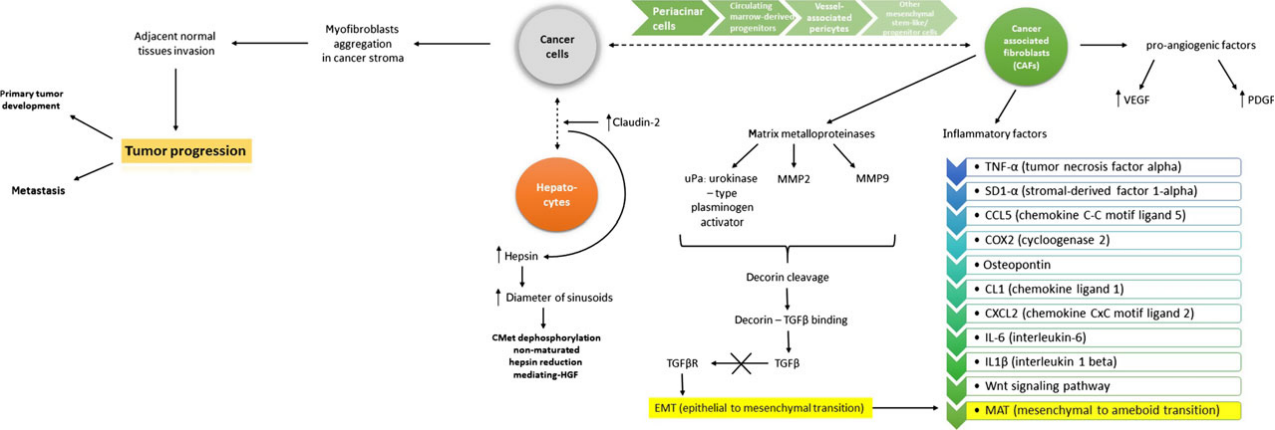
Another mechanism of interaction between cancer cells and hepatocytes is connected with claudin-2 up-regulation (which induces this interaction) and—even more importantly—hepsin. The up-regulation of hepsin causes the liver sinusoids to become bigger in size. The higher sinusoid diameter, the lower probability of metastases formation (one of probable theories in this matter, among others, for instance the ‘seed and soil’ hypothesis). In the interaction process between cancer cells and cancer-associated fibroblasts, many crucial factors play its role: pro-angiogenic factors (VEGF: vascular epithelial growth factor and PDGF: platelet-derived growth factor), inflammatory factors showed and explained on the figure, and finally matrix metalloproteinases uPa (urokinase-type plasminogen activator, as well as matrix metalloproteinases 2 and 9). All of the latter lead to the disruption of TGFβ (transforming growth factor beta) binding with its receptor, which in turn leads to EMT (mentioned previously). Interestingly, subsequently after EMT, MET occurs (mesenchymal to amoeboid transition).

Enhanced expression of this pro-inflammatory gene indicates that CAFs play an important role throughout the entire process of carcinogenesis, resulting in proliferation promotion, migration and invasion of tumour cells 56. Intriguingly, normal fibroblasts can be ‘educated’ by carcinoma cells to express pro-inflammatory genes to promote tumour development. This ‘education’ seems to be reversible 20. CAFs have also the capacity to modulate the host's immune system to promote primary and secondary tumour growth 57–60. Moreover, physical stimulation of CAFs and/or their ECM changes gene expression and the secretory pattern of CAFs favouring pro-inflammatory gene expression. This in turn helps with angiogenesis, invasion, metastasis and also apoptosis 61–63. The mechanisms connected with CAF–cancer cell interaction are presented on Figure1, as well as those related to hepatocyte–cancer cell interaction, described in the chapter below.

Cancer-associated fibroblasts role in tumour burden has been extensively investigated, thus the knowledge regarding this type of stromal cell is the broadest compared to other types of TME cells. However, the contribution of other cells is also fundamental.

## Cancer cell–hepatocyte interaction in cancer progression

Hepatic tissue is a primary target for metastases for most disseminating cancers 64. Liver structure has an impact on developing metastasis, a process which begins with the retention of circulating cancer cells in the liver sinusoids. The sinusoid diameter is critical for cancer cell invasion and plays an important role in establishment of secondary tumours 65. The size of liver sinusoids is determined by the expression of the serine protease hepsin. Hepsin is cell surface serine protease and located primarily in the plasma membrane with its trypsin-type protease module (the C-terminal half) at the external surface of cells. This enzyme is produced in most tissues, but abundantly expressed on the surface of hepatocytes 66. *In vitro* and *in vivo* studies showed enhanced hepsin expression in breast cancer tissues, which is associated with tumour growth and progression 67. On the other hand, in gastric cancer hepsin expression is decreased and predicts a poor prognosis 68. Liver sinusoids from knockout hepsin−/− mice were significantly narrower than those from the wild-type (WT). A significant decrease in the diameter of the liver sinusoids was observed with anti-hepsin treatment of the WT, which confirms that expression of hepsin affects the diameter of liver sinusoids 69. The initial step in liver metastasis is a physical trapping of circulating cancer cells in the liver sinusoids indicating that their size determines this process. Studies showed a greater number of tumour cells in the hepsin−/− liver sinusoids than in the WT liver sinusoids. Moreover, in correlation with the preferential retention of metastatic tumour cells, there was a seven-fold increase in the number of tumour colonies in hepsin−/− mouse livers in comparison with the WT mice. These results strongly suggest that loss of hepsin enhances colonization of livers by tumour cells, probably through increased retention of tumour cells associated with narrower sinusoids 69. The proposed mechanism underlying the observed phenomenon is hepsin reduction mediating hepatocyte growth factor maturation and downstream c-Met phosphorylation required for expressing proper levels of connexins. These are critical for the maintenance of normal hepatocyte size and ultimately normal sinusoidal diameter 69. In agreement with these results are findings of a lower level of hepsin mRNA in liver *versus* bone and lymph node secondary tumours of prostate cancer 70.

However, data about the role of hepsin in formation of liver metastases remain controversial. The contribution of hepsin in the development of secondary liver tumours is confirmed by the results of studies conducted on prostate cancer models 71. In contrast to Hsu *et al*. (2012), Klezovitch *et al*. 71 found that active hepsin in metastatic lesions and its up-regulation is responsible for liver metastasis. The authors found that up-regulation of hepsin resulted in marked progression of prostate tumours causing development of metastasis towards the liver. Klezovitch *et al*. did not investigate the pathway and molecular mechanism involved in hepsin mediation of hepatic metastasis development. Further investigations would explain the discrepancies in the results obtained between these two groups. Hence, the hepsin level may not be a proper prediction factor for hepatic metastasis.

In liver metastases of breast cancer, claudin-2 expression was found to be elevated in comparison to primary tumour 72,73. In kidney proximal tubule, claudin-2 is expressed in high level 74. Haplo-insufficiency of claudin-2 gene mice has reduced reabsorption of Na^+^ in the proximal tubule. Similar results have been obtained with *in vitro* cultured cells 75. Claudin-2 was found to be one of the factors involved in interactions of cancer cells with cells of the target tissue determining the metastatic spread. In contrast to liver metastases this protein is not prevalent in bone or lung metastases, confirming that claudin-2 promoted cell–cell interplay between cancer cells and hepatocytes 73. Claudin-2 functions at an early stage following the seeding of the liver to promote breast cancer cell colonization and metastatic formation. Attachment assays confirmed that diminished claudin-2 expression levels resulted in significant reduction in the ability of cancer cells to adhere to hepatocytes 73. It was also reported that claudin-2 expression may be lost during certain stages of the metastatic process, such as early dissemination from the primary tumour. Claudin-2 probably is unregulated in response to signals coming from the liver microenvironment and its expression is likely regained at later stages of metastasis 73. The authors suggest that the formation of claudin-2-mediated cell–cell interactions between cancer cells and hepatocytes may entail induction of c-Met and in turn activate the pathways responsible for development of metastasis 73.

Colon cancer patients with high levels of metallopeptidase inhibitor 1 (TIMP-1) detected at the time of their initial surgery were found to have a high risk of metachronous liver metastasis and hepatic recurrence following resection of synchronous liver metastasis. Levels of TIMP-1 were found to be significant predictive factors for poor prognosis following liver resection. The authors claim that validation of the obtained results may provide a greater understanding of colon cancer liver metastasis 76. Nevertheless, the perturbation of the TIMP-1 level is common for most inflammatory diseases and as inflammatory factors play a major role in mediating tumourigenesis, its fluctuating level does not seem to be a unique marker for liver metastasis 77–80. On the other hand, monitoring the level of TIMP-1 can be a useful tool for monitoring disease progression.

A study of single-nucleotide polymorphisms (SNPs) in colorectal primary tumours with liver metastasis detected recurrent breakpoints at chromosome 17p restricted to the *FAM27L* gene, whose function is unknown. Thus, it is likely that disruption of the *FAM27L* gene may play a role in the malignant transformation and/or the metastasis of collateral tumours into the liver 81. Further examination of sporadic colorectal cancer liver metastases *versus* primary tumours demonstrated that hepatic metastases showed acquisition of genetic aberrations that were not found in their paired primary tumours 82. These new aberrations mainly consisted of increased frequency of genetic lesions of chromosomes that had previously been associated with metastatic colorectal carcinoma (1p, 7p, 8q, 13q, 17p, 18q, 20q) and acquisition of new chromosomal abnormalities (*e.g*. losses of chromosomes 4 and 10q and gains of chromosomes 5p and 6p). These genetic changes may be the result of the metastatic process and/or adaption of metastatic cells to the liver microenvironment 82. Among the genes on aberrant chromosomes, *TWIST1* plays an important role in liver metastasis because of its influence on angiogenesis 82. Consistent with these results are studies of Mironchik *et al*. (2005) demonstrating that *TWIST1* overexpression induces *in vivo* angiogenesis and correlates with chromosomal instability in breast cancer 83.

Yet, these findings are in contradiction with the results of Malfettone *et al*. 84 who reported lower expression of *TWIST1* in hepatic secondary tumours, speculating that *TWIST1* is involved in an early step of metastasis when it is highly expressed 84. Observed lower expression of *TWIST1* could be explained by the fact that analysis was conducted on samples from already existing liver metastases when the process had already occurred 84. The same group found that the Na^+^/H^+^ exchanger regulating factor 1 (NHERF1) correlated positively with hypoxia inducible factor 1α (HIF-1α) 84. These preliminary studies on tissue sample cohorts from metastatic sites of colorectal cancer compared distant and adjacent normal mucosa and those in liver metastases indicated that nuclear NHERF1 as well as nuclear HIF-1α are expressed at a significantly higher lever, which could be related to the hostile, hypoxic TME, favouring a more invasive phenotype 84.

Research has linked obesity to hepatic metastasis, and insulin-like growth factor-I (IGF-I) plays a key role in the mechanism underlying this phenomenon. Signalling by IGF-I is responsible for establishment of hepatic metastases 85. IGF-I promotes liver metastasis not only through a direct paracrine effect on tumour cell survival and proliferation but also through indirect effects, likely involving the host microenvironment and pro-inflammatory responses 85. Nevertheless, the crosstalk between the cancer cell and hepatocyte interaction in cancer progression is still an unresolved issue and further investigations are necessary.

## Cancer cell–lung epithelial cell interaction in cancer progression

Gene expression analysis in adenocarcinoma, squamous cell carcinoma (SCC) and normal bronchus epithelium without metaplasia or dysplasia derived from patients with neoplastic changes revealed 43 differentially expressed genes 86. It is worth emphasizing that representative normal bronchus tissues were selected, containing the putative lung cancer precursor cells. Among the investigated genes were key regulators of biological functions, including five epithelium related adhesion genes, two subunits of integrin receptors (*ITGA3* and *ITGB4*) and three genes involved in the desmosome complex (*DSP*, *plakoglobin* and *DSC3*) 86. In contrast to explants from normal bronchus and adenocarcinoma, SCC showed abundant RNA and protein expression of DSP, plakoglobin and DSC3 depending on the localization (specific central and peripheral localization). The distribution of these desmosome molecules is very similar to the pattern in normal squamous epithelium of the skin indicating a normal expression regulation of these molecules in malignant transformed SCC and may have implications for the biological behaviour of tumour cells 86.

The direct physical contact between normal mesenchymal cells and lung carcinoma cells inhibited growth of malignant cells *in vitro* 87. Co-culture of normal rat tracheal epithelial cells with chemically transformed tracheal epithelial cells suppressed the tumourigenicity of these cells upon inoculation into denuded tracheal grafts 88 89. The strong presence of these molecules in SCC and its normal distribution may be associated with the less frequent and late metastasis pattern of SCC as compared with adenocarcinoma. Nevertheless, this conclusion needs further investigation 86. Furthermore, factors physiologically released by bronchial epithelium such as TGF-β, epidermal growth factor (EGF) and bombesin contribute to tumour growth 90–93. EGF and TGF-β affect gap junction communication which is observed in tracheobronchial epithelium as these molecules can influence communication between epithelial cells during cancer growth 89,94,95. In comparison to bronchial epithelium, SCC secretes much more transforming growth factor α, and expressed greater numbers of cross-reacting EGF receptors. This in turn acts in an autocrine and paracrine manner increasing epidermal growth factor receptor (EGFR) 96. Taking into consideration enhanced EGFR expression and a high physiological level of EGF, this confirms interplay between cancer cells and epithelial lung cells in determining cancer development. Surprisingly, co-culture of chemically transformed tracheal epithelial cells with normal rat tracheal epithelial cells suppressed tumourigenicity of these cells upon inoculation into denuded tracheal grafts 88 suggesting that tumourigenic potential may be related to the biological isolation of a sufficiently large focus of transformed cells from normal cells 89.

Mennerich *et al*. observed enhanced expression of syndecan-1 (*SDC-1*) in bronchial carcinoma compared to normal tissue 97. This enhanced level of *SDC-1* was detected in stromal cells and probably was expressed in epithelial cells on account of their dominance in stroma. Marker analysis suggested a shift of *SDC*-1 expression from epithelial tumour cells to myofibroblasts within the connective tissue surrounding the tumour cells 97. The shift of *SDC-1* expression from tumour to stromal cells may be associated with a decreased expression within epithelial tumour cells and it can have important consequences 97. *SDC-1* down regulation potentially generates the ability to form a dedifferentiated invasive or migrating tumour cell, mobilization of growth factors from epithelial tumour cell surfaces and ECM and soluble factors needed for the establishment of the cancer-associated non-malignant stroma 97,98. The above findings support the hypothesis that the cancer-associated non-malignant stromal cells contribute to tumour cell invasion and the development of metastases 97.

## Interaction between cancer cells and normal cells of the primary organ

Hyaluronan (HA) concentration in cells is enhanced under pathological conditions and disturbs the normal cell–cell and cell–ECM interactions in simple epithelia, leading to aberrant epithelial morphogenesis. These morphological abnormalities *in vitro* in a kidney model upon stimulated HA synthase 3 gene expression may be related to pre-malignant changes, including intraluminal invasion and deregulated epithelialization, probably mediated by mitotic spindle orientation defects 99. The results indicate that cancer cells may directly or indirectly secrete HA which is in agreement with previously studies 100,101. In this manner, increased HA concentration in the ‘healthy’ environment interferes with cell–cell adhesions, resulting in the disruption of epithelial barrier function and pre-neoplastic changes.

*In vitro* observation made by Pistone *et al*. 102 that conditioned media (CM) collected from cancer increases the proliferation of non-tumour mammary epithelial cell line. The authors conclude that this phenomenon is because of the presence of soluble factors in CM derived from the tumour cell line, which are present in CM and absent in the healthy one 102. This evidence suggests possibility that a tumour is able to favour tumour growth through secreted molecules modifying the normal microenvironment.

One of the factors taking part in this interplay may be IL-15. It has been proposed recombinant IL-15 may kill tumour cells by stopping the blood flow to the tumour and by stimulating white blood cells to kill kidney cancer cells (according to clinical trial NCT01727076). On the basis of observations made on a renal cancer model, IL-15 initiates the EMT process in tumoural and paratumoural cell lines in contrast to normal cells where it inhibits the drift towards EMT 103. On the other hand, Spink *et al*. obtained evidence against these findings 104. Co-culture of normal and cancer cell lines, the same as used by Pistone *et al*., revealed that normal epithelial cells inhibit tumour cell proliferation and demonstrated the utility of this co-culture system as a model of early paracrine control of breast cancer 102,104. IL-15 is also known to be connected with the Wnt signalling pathway 105. Results obtained on a rheumatoid arthritis fibroblast-like synoviocyte model revealed that expression of Wnt influences expression of IL-15 105. A similar link between these two may exist in neoplastic cells as well. In a colon cancer model, not only does Wnt initiate a plethora of cell-intrinsic alterations in epithelial cells, but it also impacts on how epithelial cells communicate with macrophages 106. Inactivation of the β-catenin allele in the colonic epithelial cell line HCT116 significantly altered its interaction with macrophages. In fact, macrophages failed to promote Wnt signalling and to protect HCT116 WT cells from apoptosis. This is consistent with an earlier report stating that the expression of dnTCF4 in tumour cells prevented signalling between macrophages and the tumour 107,108. Moreover, production of IL-15 by colon cancer cells was associated with depletion of tumour-associated macrophages (TAMs) 109. All these studies demonstrate that acquisition of oncogenic mutations alters the interaction of epithelial cells with the adjacent stroma. Activation of oncogenic pathways, such as Wnt, not only triggers cell-intrinsic changes but also induces stromal alterations that are required for tumour progression 106.

Wnt also shows a link to the inducible nitric oxide synthase (iNOS) and Src kinase 110,111. Both iNOS and Src are involved in tumour progression and apoptosis 112,113. In both normal and cancer cells, iNOS is regulated by Src kinase 114. It is possible that tumour cells secreting pro-inflammatory cytokines, such as IL-15 which also enhances nitric oxide production, may influence phosphorylation of iNOS by Src in lung tissue epithelium 114,115. That in turn plays an important role in the regulation of iNOS and nitric oxide production and hence could account for some Src-related roles in inflammation and cancer 114.

In agreement with the cited results presented above, we suggest that it is not the predisposition of the normal tissue which allows for secondary tumour formation, but the ability to properly respond to factors secreted by neoplastic cells that is most crucial here. The response manifests itself in the adjustment of the secretome, the proteome, the transcriptome, the expression of cell surface molecules and the deregulation of signal transduction pathways, all of which favour metastasis.

## Cancer cell–pleural and peritoneal cells interaction in cancer progression

**Fig 2:**
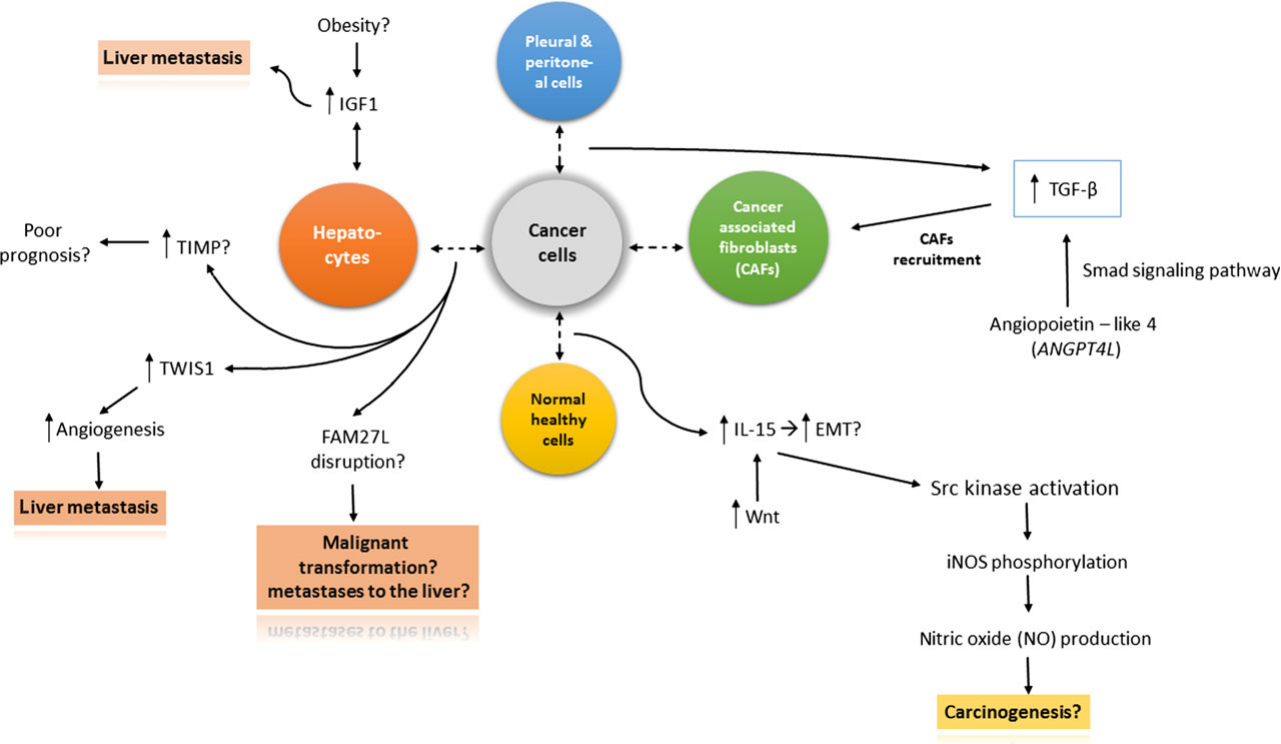
The figure shows interaction mechanisms between cancer cells and: (1) Hepatocytes: overexpression of TIMP (Tissue inhibitor of metalloproteinase) is presumed to cause poor prognosis in patients. At the same time, TWIST1 (Twist Basic Helix–Loop–Helix Transcription Factor 1) and FAM27L (Family With Sequence Similarity 27-Like) expression disruption, as well as the same probably in case of insulin growth factor 1 (IGF-1) may lead to metastases formation. (2) Normal healthy cells: overexpression of Wnt protein causes the up-regulation of IL-15 (interleukin-15), which is the probable cause of the excessive epithelial to mesenchymal transition (EMT). After several subsequent processes, the process of carcinogenesis is presumably induced. (3) Interactions between cancer cells and pleural/peritoneal cells, as well as between cancer cells and cancer-associated fibroblasts (CAFs) have one significant molecule in common—it is TGF-β (transforming growth factor beta), whose overexpression leads to CAFs recruitment. The main probable reasons of TGF-β overexpression is the expression of angiopoietin-like 4 (ANGPT4L) which acts *via* Smad signalling pathway.

Limited data are currently available on factors associated with pleural and peritoneal metastasis, even though malignant mesothelioma, *i.e*. the primary tumour arising from mesothelial surface, could be considered as a specific model for such interaction. Those have been presented on Figure2, together with the factors associated with metastases to lung and interaction with normal cells. Unfortunately, some peculiarities of malignant mesothelioma (*e.g*. its asbestos-related pathogenesis, or the putative role of polioma virus SV40 in enhancing asbestos-related tumourigenesis) make the results achieved so far in this tumour hardly transferable in other solid neoplasms metastasizing to the pleural surface. However, it is also clear in case of this tumour that cancer cells instigate tumour-associated fibroblasts, promoting tumour progression *via* a malignant cytokine network 116.

In matched primary breast cancers and pleural effusions (PE), MMP-2 levels were found to be elevated at metastatic sites 117. MMP-2 is considered as a biomarker in many other cancer types including retinoblastoma 118, colon cancer 119, lymphoma 120, thyroid cancer 121, lung cancer 122, glioblastoma multiforme 123, gastric cancer 124. MMP-2 does not seem to be specific or unique. Thus, there is an urgent need to investigate other potential factors involved in this process. However, in oesophageal SCC, MMP-2 is regulated *in vivo* and *in vitro inter alia* by S100A4 (positively) and E-cadherin (negatively), these are important factors involved in control of invasion and metastasis 125.

A large body of evidence supports the calcium-binding protein S100A4 role in control of invasion and metastasis in many invasive tumours. For example, cDNA microarray analysis of peritoneal metastasis in gastric adenocarcinoma indicates that the S100A4 and *CTNNB1* genes encoding a subunit of E-cadherin–β-catenin were up-regulated in the examined cell lines 126. S100A4 was also found to mediate the attraction and/or activation of T cells associated with promotion of metastatic spread 58. In pulmonary metastasis, S100A4 deficiency was shown to determine low vascularity, which in turn suppresses development of secondary tumours 58. Moreover, in lung cancer patients, a mutation in exon 3 of the *CTNNB1* gene TCT [(Ser) –> TGT (Cys)] was correlated with the formation of metastatic sites 127. All this facts indicates that a mutation in the *CTNNB1* gene favours the formation of pulmonary metastases, while malfunction of the S100A4 gene may prevent from this mechanism. S100A4 also positively regulates MMP-2. Taken together mentioned interactions, also with other described factors, this data emphasize the need for further investigation of MMP-2 signal transduction pathway components to obtain information about molecules mediating pleural and peritoneal metastasis.

Observed elevated levels of SDF-1alpha/CXCL12 in pleural malignant exudates may indicate its correlation with metastasis and its possible participation in pleural trafficking in lung cancer 128. This hypothesis was confirmed by observation that cancer cells express CXCR4 in malignant PE and also mesothelial cells of the pleura stained positive for SDF-1α 129. Furthermore, the CXCR4 receptor was functionally expressed in lung cancer cell lines, where it regulates their migration, adhesion and morphological change 130. In addition, a post-transcriptional expression protein level of SDF-1α/CXCL12 was positively correlated with functional pleural fluid characteristics like lactate dehydrogenase and/or glucose. Furthermore, in malignant mesothelioma (again the prototype model of interaction of cancer cell with normal mesothelial cells), SDF-1α/CXCL12 was proved to be involved in recruitment of mesothelioma stem-like progenitor cells 131.

The connection between SDF-1α/CXCL12 and glucose metabolism may have an impact on dissemination of malignant cells into pleural space. This is in accordance with results obtained from oligonucleotide microarray on tissue sample cohorts 132. Lin *et al*. demonstrated that genes related to cellular metabolism, aldolase A, sorbitol dehydrogenase, transketolase, and tuberous sclerosis 1 are important in malignant PE pathogenesis 132. According to these data, authors concluded that glucose metabolic reprogramming may play a crucial role in pleural metastasis. On the other hand, altered expression pattern of genes related to glucose metabolism in cancer tissue samples is obviously dependent on the Warburg effect 133.

Surprisingly, lung tumours exhibiting pleural invasion also showed elevated expression of apoptosis-related markers: caspase-3 (94.5%), p53 (60%) and bcl-2 (54.5%). As these proteins show rather an anticancer function, their role in metastasis is controversial and unclear 134, as is the role of the gene encoding Semaphorin-3B (*SEMA3B*) in promotion of metastasis. Research carried out on human cancerous cells derived from tumours that metastasized to the lung revealed that paradoxically, *SEMA3B* inhibited growth of primary tumours, but induced metastasis *in vivo* 135. *SEMA3B*-expressing tumours exhibited notable defects of pericyte recruitment to blood vessels compared with control xenografts. In tumour cells, *SEMA3B* expression induced the secretion of IL-8 135, a cytokine also associated with tumour progression and metastasis as well 136. Moreover, IL-8 activity is required to mediate the recruitment of TAMs and metastatic dissemination in SEMA3B-expressing tumours. Data indicate that *SEMA3B* activity is mediated by NP1-dependent activation of p38, subsequent activation of mitogen-activated protein kinase stimulating p21 protein, which in turn inhibits cancer cell proliferation and IL-8 biosynthesis promoting invasiveness 135.

Another cytokine involved in lung metastasis is TGF-β 137. TGF-β is produced by the cancer cells themselves, macrophages and mesenchymal cells. TGF-β primes tumour cells for metastatic seeding and its signalling enhances mammary tumour dissemination to the lungs 137. Crucial to this process is the induction of angiopoietin-like 4 (*ANGPTL4*) *via* the Smad signalling pathway. Upon entering blood circulation and reaching lung capillaries, these cells secrete Angptl4 which disrupts endothelial cell junctions, facilitating more efficient transfer to the lung parenchyma 137.

Investigation of breast cancer model also revealed a specific gene expression pattern in primary tumours that predestinates them for lung metastasis. Minn *et al*. found 54 genes overexpressed in primary breast tumours that promote lung metastases as well 138. Most of these genes encode extracellular products including growth and survival factors for example the HER/ErbB receptor ligand epiregulin, chemokines (CXCL1), cell adhesion receptors like ROBO1 and extracellular proteases like MMP-1. Minn *et al*. have also included intracellular enzymes like COX2 and transcriptional regulators like Id1.

These genes encode the epidermal growth factor family member epiregulin (EREG), MMP2, MMP1, the chemokine GRO1/CXCL1, the cell adhesion molecule SPARC, and the cell adhesion receptor VCAM1 138. It is worth noting as well that these genes seem to be not only markers but also functional mediators of lung-specific metastasis 137,139,140. Inhibition of EGFR and COX2 can decrease the lung metastatic progression in a clinically relevant model of breast cancer 140. The *Id1* gene encoding a transcriptional regulator and its expression in breast cancer cells has a significant impact on the ability of breast cancer cells to metastasize to the lungs in xenograft models 140. Further investigations revealed that the expression of *Id1, CXCL1*, *COX2*, *EREG*, and *MMP1* mRNAs increases with lung metastatic ability but is not restricted to aggressive lung metastasis populations. The protein levels were correlated with mRNA levels 138. However, additional analyses indicated that only cells overexpressing Id1 alone were slightly more active at forming lung metastases than cells infected with vector controls 140. Considering these data, one may assume that perturbation in Id1 expression/function may entail other aberration in activity of CXCL1, COX2, EREG and MMP1 resulting in the metastatic proclivity of a tumour.

Research into genes that predestine the development of pleural metastases has led to the finding of a set of genes named Lung Metastasis Signature (LMS). LMS includes 18 genes encoding cell surface proteins and secreted products that affect the interaction of tumour cells with the microenvironment 140. Previously mentioned EREG, COX2, MMP1 and MMP2 are a subset of LMS genes that reconstitute a multi-functional vascular remodelling programme which promotes metastatic progression. This set of genes determines lung vasculature branching and enables extravasation of cancer cells on their dissemination from mammary tumours to the lungs. In addition, LMS causes inhibition of EGFR and COX2, which in turn, decrease lung metastatic progression in a clinically relevant model of breast cancer 139. Nevertheless, upon retroviral infection, only cells overexpressing Id1 alone were slightly more active at forming lung metastases than cells infected with vector controls which indicates its crucial function in metastasis 138. Indeed immunohistochemistry results demonstrate that emergent lung metastases also express abundant levels of nuclear Id1 in a subset of tumour cells within these lesions 140. However, the enforced overexpression of Id1 alone is not sufficient to render parental MDA-MB-231 cells efficiently metastatic to the lungs 138.

It appears that not only Id1 but also Id3 is involved in this process 140. Interestingly, there were statistically significant effects of individual Id1 and Id3 knock-down on decrease in lung metastatic outgrowth, where the combined Id1/Id3 knock-down resulted in complete suppression of lung metastatic colonization suggesting that both Id1 and Id3 proteins are required for lung metastasis 140.

In 2008, Landemaine *et al*. 141 described another new set of six genes that contribute to lung metastasis: *DSC2*, *TFCP2L1*, *UGT8*, *ITGB8*, *ANP32E* and *FERMT1*. These encode adhesion molecules that belong to various protein families including desmosome proteins (DSC2), integrins (ITGB8) and focal adhesion molecules (FERMT1); molecules involved in regulation of transcription (TFCP2L1), glycosphingolipid biosynthesis (UGT8) and phosphatase inhibitor activity (ANP32E) 141. Hence, it is difficult to ascertain exactly which should belong to the LMS list.

Tumour cells exhibit a selective attachment pattern and growth preference on specific sites within peritoneal tissues 142. Tumour cells are localized specifically to the CD45 immune aggregates and are not found in the surrounding areas of the omentum. Moreover, their growth is aggressive indicating that the vasculature in these areas may be especially conducive to supporting cell growth. Indeed, research has revealed that vessels within aggregates moderately express CD105: a proliferation-associated antigen located predominately on endothelial cells undergoing active angiogenesis, 143 in contrast to vessels outside of the aggregate which are low or negative for CD105 142. The vessels within the immune aggregates are in a pro-angiogenic state distinct from the vessels in the surrounding tissue 142. In this case, a question arises whether some features of target tissue alter the gene expression pattern of cancer cells to favour their motility, proliferation, invasiveness and growth of secondary tumours.

## Summary

**Fig 3:**
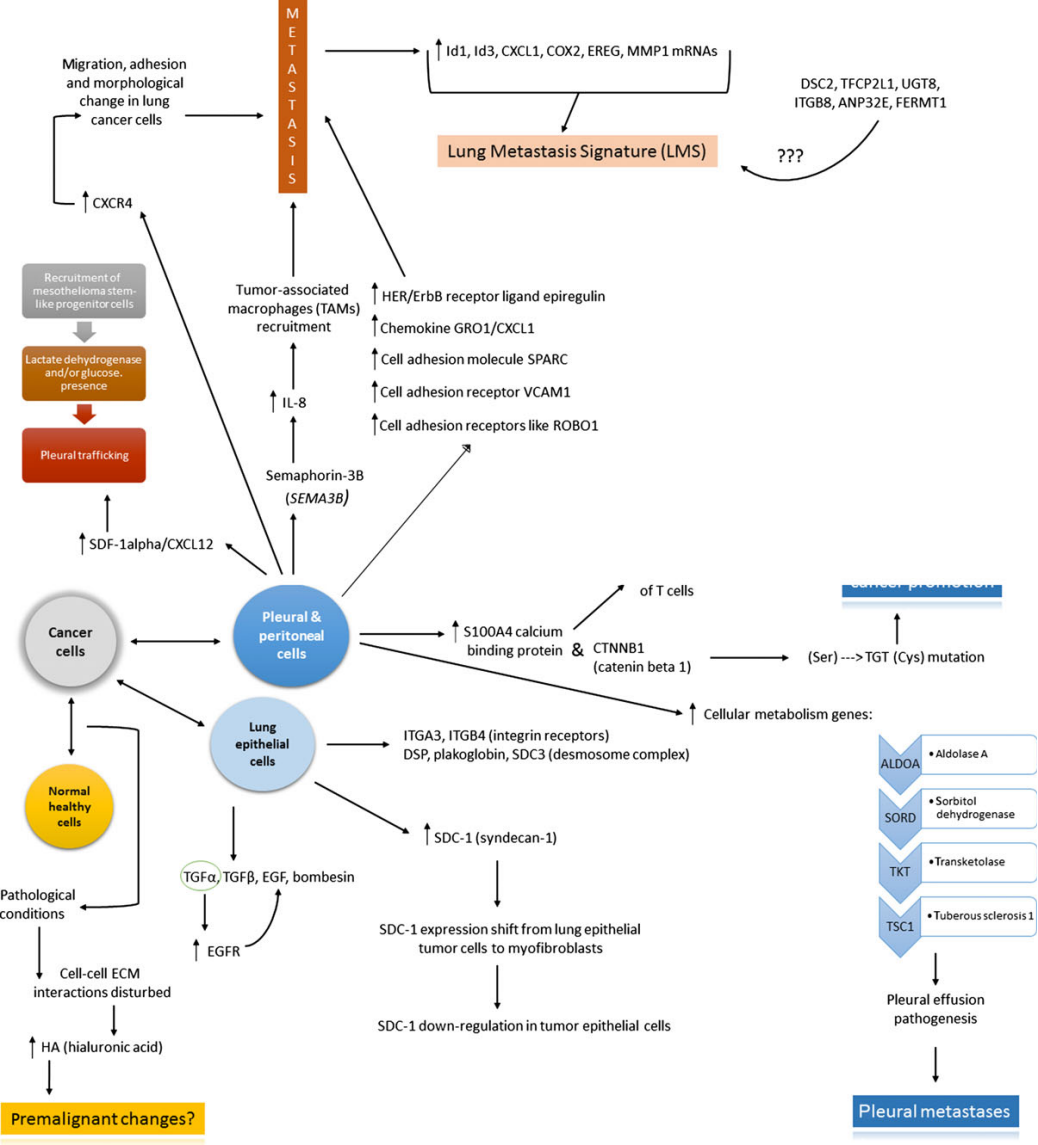
As a result of the interaction between normal healthy cells and cancer cells, it is possible that under pathological conditions, interactions between normal cells and its extracellular matrix (ECM) become disrupted. This in turn causes *inter alia* the overexpression of hyaluronic acid which has been shown in several publications to be probably responsible for pre-malignant changes. Concerning the interactions between cancer cells and lung epithelial cells, there are several factors which differently but altogether lead to tumour cell invasion and metastases. Among them, there are ITGA3, ITGB4 (integrin receptors A3 and B4), DSP (desmoplakin), plakoglobin and SDC3 (desmosome complex 3) which act directly. On the other hand, SDC-1 (syndecan-1) becomes down-regulated to cause the same process, while TGFα, TGFβ (transforming growth factors alpha and beta), EGF (epidermal growth factor) and bombesin act through epidermal growth factor receptor (EGFR), forming a ‘vicious circle’: TGFα causes EGFR up-regulation, which in turn causes TGFβ, EGF and bombesin overexpression. This loop results simultaneously in metastases formation. Finally, cancer cells interact with pleural and peritoneal cells *via* overexpression of S100A4 calcium-binding protein (resulting in the attraction and activation of T cells) and CTNNB1 (catenin beta 1 – causes frameshift mutation which results in cysteine instead of serine production). The final step in each case is mostly cancer promotion.

It is well-established that the TME affects tumour growth and metastasis 144. The overall view on interactions between tumour cells and other types of cells is presented on Figure3. A growing body of evidence indicates that direct interactions between healthy/normal cells and neoplastic ones contribute to tumour growth as well. Hence, in recent years researchers expanded the field of interest in cancer study to surrounding cells and their interactions with cancer cells and this topic has become the focus of intense research. Looking ahead, our growing understanding of the alterations that occur in the stromal cells in TME might prove useful in prognosis and generate new therapeutic targets.
